# Long-Term Outcome of Brachial Plexus Reimplantation After Complete Brachial Plexus Avulsion Injury

**DOI:** 10.1016/j.wneu.2017.03.052

**Published:** 2017-07

**Authors:** Carolina Kachramanoglou, Thomas Carlstedt, Martin Koltzenburg, David Choi

**Affiliations:** Spinal Repair Unit, UCL Institute of Neurology, London, England, United Kingdom

**Keywords:** Brachial plexus, Brachial plexus avulsion, Brachial plexus reimplantation surgery, BP, Brachial plexus, CMAP, Compound muscle action potential, DASH, Disabilities of the Arm, Shoulder, and Hand, EMG, Electromyelography, FDS, Flexor digitorum superficialis, MRC, Medical Research Council, MUAP, Motor unit action potential, PSW, Positive sharp waves, SCV, Sensory conduction velocity, SD, Standard deviation, SF-36, Short Form-36, SNAP, Sensory nerve action potential

## Abstract

**Background:**

Complete brachial plexus avulsion injury is a severe disabling injury due to traction to the brachial plexus. Brachial plexus reimplantation is an emerging surgical technique for the management of complete brachial plexus avulsion injury.

**Objective:**

We assessed the functional recovery in 15 patients who underwent brachial plexus reimplantation surgery after complete brachial plexus avulsion injury with clinical examination and electrophysiological testing.

**Methods:**

We included all patients who underwent brachial plexus reimplantation in our institution between 1997 and 2010. Patients were assessed with detailed motor and sensory clinical examination and motor and sensory electrophysiological tests.

**Results:**

We found that patients who had reimplantation surgery demonstrated an improvement in Medical Research Council power in the deltoid, pectoralis, and infraspinatous muscles and global Medical Research Council score. Eight patients achieved at least grade 3 MRC power in at least one muscle group of the arm. Improved reinnervation by electromyelography criteria was found in infraspinatous, biceps, and triceps muscles. There was evidence of ongoing innervation in 3 patients. Sensory testing in affected dermatomes also showed better recovery at C5, C6, and T1 dermatomes. The best recovery was seen in the C5 dermatome.

**Conclusions:**

Our results demonstrate a definite but limited improvement in motor and sensory recovery after reimplantation surgery in patients with complete brachial plexus injury. We hypothesize that further improvement may be achieved by using regenerative cell technologies at the time of repair.

## Introduction

Avulsion of one or more roots is seen clinically in approximately 70% of severe brachial plexus (BP) traction injuries. Complete BP avulsion injury is a severe, disabling injury, predominately affecting young men in high-energy motorcycle accidents due to traction to the BP when the rider falls on the shoulder.^1^

Historically, attempts to restore function were limited to nerve transfers. Nerve transfers involve the sacrifice of the function of a lesser-valued donor muscle to revive function in the recipient nerve and muscle, with subsequent reinnervation.^2^ Nerve-transfer techniques allow return of some function, but the overall recovery remains poor.^3^, ^4^, ^5^ In recent years, BP reimplantation has been introduced and offers an alternative surgical strategy for the treatment of BP avulsion injury.^6^, ^7^, ^8^, ^9^, ^10^, ^11^, ^12^, ^13^ This operation involves the implantation of avulsed ventral roots into the anterolateral aspect of the spinal cord.^10^ Regenerating motor fibers travel through the reimplanted nerve roots to reinnervate target muscles.^6^, ^8^, ^14^, ^15^, ^16^, ^17^, ^18^, ^19^, ^20^

The aim of this observational study was to assess the degree of functional recovery in the affected arm of patients who have undergone BP reimplantation surgery after complete (C5 through to T1 nerve roots) BP avulsion injury. The motor and sensory functions were assessed both clinically and by the use of electrophysiological tests. Patient satisfaction and experience was assessed with the use of patient-reported questionnaires.

## Methods

The surgical procedure is accepted as a standard of care in the National Health Service, and outcome assessment was performed as routine clinical care.

### Patient Selection

Patients were identified retrospectively after inspection of the surgical records and a log provided by the surgeons for complete BP injury and BP reimplantation procedures. Initially, hospital records were reviewed for evidence of a completely paralyzed arm, complete loss of sensation from C5 to T1, evidence of the Tinel sign, and Horner syndrome at presentation. The diagnosis of complete BP avulsion was confirmed by open exploration of the BP. Correlation also was made with other investigations when available, including preoperative computed tomography myelography and preoperative electrophysiology tests.

The indication for BP reimplantation surgery was evidence of complete (C5–T1) BP injury for which alternative treatment was not available, recommended, or deemed to result in a significant neurologic improvement. The reimplantation procedure was performed as soon as possible after the time of injury and within 4 weeks. Delays were sometimes encountered as the result of multiple injuries requiring more urgent management, time for transfer to our unit, bed availability, and the patients' overall clinical condition.

### BP Reimplantation Procedure

The reimplantation procedure was performed as described in Carlsted et al.^8^, ^10^ In brief, the patient was placed in the lateral position with the affected side up and the head held in a Mayfield clamp with the neck slightly flexed. The operating table was positioned 15° head up to minimize venous congestion and bleeding. A supraclavicular skin incision was made and extended laterally in parallel to the clavicle and cranially in a vertical line towards the mastoid process. The spinal accessory nerve was identified and protected as it emerged from the dorsal aspect of the upper part of the sternocleidomastoid muscle. The BP was then identified and dissected. Subsequently, the lateral masses of C5–C7 and transverse process of T1 were approached between the levator scapulae and the posterior and medial scalenus muscles, and the longissimus muscle was split longitudinally to approach the spine. The paravertebral muscles were dissected from the hemilaminae and C5–C7 hemilaminectomy, and medial one-third facetectomy was performed. The denticulate ligaments were cut and held by stay sutures, and the spinal cord was rotated gently to bring its ventrolateral aspect into view.

A nerve graft was taken from the superficial radial nerve or medial cutaneous nerve of the forearm. The avulsed C5–T1 roots were trimmed distally to the level of normal-appearing nerve root or to the junction with the ventral root in an attempt to remove the dorsal root ganglia. The nerve grafts were stitched to the avulsed roots, retrieved through or around the intervertebral foramina, and implanted into the spinal cord by making 2–3 mm longitudinal slits in the pia mater of the spinal cord, as close as possible to the ventral root exit zone. The grafts are positioned 1–2 mm deep to the pia mater in the spinal cord and the retained by the use of fibrin glue around the outside of the nerve sheath and pia of the spinal cord. Spinal cord monitoring was performed throughout the procedure to avoid an injury to the spinal cord, particularly when tilted and during reimplantation of the roots. No perioperative or postoperative complications related to the surgical procedure were observed.

### Motor and Sensory Clinical Assessment

All patients were assessed clinically based on the Medical Research Council (MRC) scale to estimate limb and axial muscle strength. A summated muscle score based on the MRC clinical scale also was used to assess global power in the affected arm (“global MRC score”). This was obtained by assessing 7 upper limb muscles or muscle groups for MRC motor power. Muscles assessed were the deltoid (C5–C6 root values)/supraspinatous (C4–C6), infraspinatous (C5–C6), pectoralis (C5–C6), biceps brachii (C5–C6), triceps (C6–C8); for wrist movements extensor carpi radialis (C5–C6) and ulnaris (C7–C8)/flexor carpi radialis (C6–C7) and ulnaris (C7–C8, T1); and for finger movements the flexor digitorum superficialis (C7–C8, T1) and profundus (C7–C8, T1)/flexor digiti minimi (C7–C8, T1)/flexor pollicis (C8–T1)/extensor digitorum (C7–C8)/extensor indicis (C7–C8)/extensor pollicis brevis (C8–T1) and longus (C7–C8, T1) interossei (C8–T1). MRC scores for each muscle/muscle groups were then added together to obtain the “global MRC score,” ranging from 0 to 35.

All patients underwent sensory testing, which included 1) light touch using cotton wool; 2) pinprick with a blunt pin; 3) vibration sense with the use of a 128-Hz tuning fork; 4) proprioception; and 5) cold temperature sense with a tuning fork at room temperature and tested 3 times. These were tested clinically at the shoulder, elbow, and wrist. The patient was asked to close his eyes during the examination. The unaffected arm was examined first. In addition, the presence of Horner syndrome was documented and the Tinel sign was tested.

### Outcome Measures

To gain an insight into the way patients perceive their health and the impact of their disability to their quality of life, 4 validated patient-reported outcome measures were used. Patients completed these questionnaires independently. The validated patient-reported outcome measures used are described in the paragraphs to follow.

First, the visual analog scale (VAS) was used to assess the severity of pain. If a patient reported referred sensations, defined as sensations that are perceived to emanate from other areas of the body distinct from the body part being stimulated, a more detailed examination was conducted, and patients were asked to describe the sensation and location to the best of their ability. Perceived sensations were drawn on a schematic diagram of the arm. Patients were told that the sensitivity of the upper arm was assessed and were not informed of the possibility of experiencing abnormal or referred sensations. Similarly, when patients reported an insensate area within a dermatome, a more careful examination was performed in an attempt to localize the insensate region, which was drawn on a schematic diagram of the arm. Finally, observations of allodynia also were recorded, defined as pain caused by stimuli that were non-noxious in the intact contralateral limb or normal subjects.

To assess the global function of the arm, the Disabilities of the Arm, Shoulder, and Hand (DASH) questionnaire was used^21^; greater DASH scores reflect greater disability. In addition, the Michigan Hand Outcomes Questionnaire was used to measure a person's perception of their hands in terms of function, appearance, and pain. Greater scores indicate better performance in all domains except pain.^22^, ^23^ Finally, the Short Form-36 (SF-36), a generic, multipurpose, short-form health survey comparing the relative burden of diseases, was completed.^24^

### Electrophysiological Assessment

Neurophysiological studies were performed in 13 of the 15 patients (86.6%) who had reimplantation surgery. The remaining 2 patients refused or did not attend the electrophysiological studies. The neurophysiological examination was performed in all patients by an experienced EMG neurophysiologist, who was blinded to the status of the patients.

All neurophysiological tests were performed with a Viking NCS, EMG, EP, IOM System (CareFusion, San Diego, California, USA; Version 12) and stimulator Digitimer DS5 (Digitimer, Welwyn Garden City, United Kingdom).

The median and ulnar nerve motor conduction data were determined via surface electrodes for stimulation at the elbow and proximal to the wrist and for recording over the abductor pollicis brevis muscle and over the abductor digiti minimi muscle. For each nerve, the following conduction data were recorded: distal motor latency, conduction velocity, amplitude, and F-wave latency stimulated at the wrist.

Sensory conduction velocity (SCV) of the median, ulnar, and radial nerves also was assessed. SCV of the median nerve was determined orthodromically from 1) the second finger, 2) the third finger, and 3) the palm to the wrist. The SCV of the ulnar nerve was tested from 1) the fifth finger and 2) the palm to the wrist and the SCV of the radial nerve was tested from the forearm to the wrist.

Needle electromyography (EMG) was performed with a TECA Elite Disposable Concentric Needle Electrode (37 mm, 26G; CareFusion, Middleton, Wisconsin, USA) and conventional EMG recorder, Viking Select EMG machine (CareFusion, San Diego, California, USA; Version 12). EMG was attempted in the following muscles: deltoid, biceps, triceps, infraspinatous, first dorsal interosseous, flexor digitorum superficialis (FDS), and extensor digitorum communis. After needle insertion, pathologic spontaneous activity, shown as fibrillation potentials and positive sharp waves (PSW), were first determined. These were rated as “0” no fibrillation potentials/PSWs, “+1”, “+2,” and “+3” with increasing severity.

The patient was then asked to contract the muscle of interest to assess the presence of motor unit action potentials (MUAPs) under voluntary control and, if present, these were recorded. The duration, amplitude, degree of polyphasicity, recruitment, and interference patterns were studied. The grading of muscle reinnervation was based on the configuration of the MUAPs. Reinnervation was graded as follows: “0,” no recorded MUAPs; “1,” MUAPs of increased duration/reduced amplitude/increased polyphasicity; “2,” MUAPs of increased duration/increased amplitude/increased polyphasicity; “3,” MUAPs of increased duration/normal amplitude/minimal polyphasicity; and “4,” MUAPs of minimally increased duration and amplitude/no polyphasicity. Assessment of recruitment was made at minimal muscle contraction to determine the recruitment pattern and at maximal voluntary contraction to assess the interference pattern.

### Statistical Analysis

All statistical analysis was performed with STATA statistical software, Version 11 (StataCorp LP, College Station, Texas, USA). A Wilcoxon–Mann–Whitney test (z) was used to compare parameters. A Spearman rank correlation coefficient (rho) was used to test for statistical correlations. Statistical significance was accepted at the 5% level (*P* < 0.05).

## Results

### Description of Patients

Twenty-five patients who had reimplantation surgery in our institution between 1997 and 2010 after complete BP avulsion were identified. Only patients with traumatic BP injury were included. Of these, 15 patients were included in the study. The remainder of patients was not assessed for one of the following reasons: 1) loss to follow-up (*n* = 5), 2) patients declined participation (*n* = 3), 3) have had amputation of the affected arm (*n* = 1). In addition, patients with evidence of brain or spinal cord injury on preoperative magnetic resonance imaging, suspicion of incomplete avulsion, or root rupture during the exploration procedure and any electrophysiological response in perioperative electrophysiological studies were excluded (*n* = 1).

All 15 patients were men (mean age of 32 years, standard deviation [SD], 9.71; range, 18–48 years). Thirteen reimplantation patients were natively right-handed (86.67%) and 2 were left-handed (13.33%). Of the 13 right-handed patients, 5 injured their right arm (38%) and 8 injured the left (62%). Both natively left-handed patients injured the left arm.

The median period from injury to exploration was 11.9 days (SD, 8.95; range, 1–33 days). The mean period from the day of injury to the day of reimplantation was 33.3 days (SD, 30.29 days; range, 4–138 days). Reimplantation surgery took place within 40 days from injury in all patients, except in 1 patient, who was not fit for surgery immediately and was operated 138 days after his injury. The median period from injury to exploration was 11.9 days (SD, 8.95; range, 1–33 days). The mean period from the day of injury to the day of reimplantation was 33.3 days (SD, 30.29 days; range, 4–138 days). Reimplantation surgery took place within 40 days from injury in all patients, except in 1 patient, who was not fit for surgery immediately and was operated 138 days after his injury. The median period from the day of injury to assessment for this study was 60.6 months (SD, 50.3; range, 10–155 months).

### Clinical Assessment of Motor Function

All 15 patients except one had complete loss of motor function of all myotomes innervating the arm (C5 to T1) before their reimplantation procedure. One patient had a flicker of movement in the serratus anterior muscle but no other movement in the shoulder, elbow, or wrist joints.

Of the 15 patients in the reimplantation group, movement was noted after surgery in 12 (80%) in the deltoid muscle, 10 (66.6%), and 10 (66.6%) patients in pectoralis and infraspinatous muscles, respectively, 9 (60%) in biceps, 9 (60%) in triceps, and 1 (6.6%), and 2 (13.3%) had wrist and finger movement, respectively. Average MRC scores are shown in Table 1 and individual MRC scores presented in Table 2. No significant correlation was detected between time from injury to intervention and neurologic recovery (rho = 0.18; *P* = 0.51).

**Table 1:** Upper Limb Motor Recovery

Muscles/Muscle Group	Reimplantation (Total = 15)		
	Number of Patients with Return in Function (%)	Number of Patients with Grade 3 Power (%)	Number of Patients with Grade 4 Power (%)
Deltoid	12 (80)	2 (13.3)	2 (13.3)
Infraspinatus	10 (66.6)	4 (33.3)	1 (6.6)
Pectoralis	10 (66.6)	5 (33.3)	0
Biceps brachii	9 (60)	0	1 (6.7)
Triceps	9 (60)	0	3 (20)
Wrist movements: extensor carpi radialis/ulnaris	1 (6.6)	0	0
Finger movements: FDS/FDP/FDM/FP/ED/EI/EPB/EPL	2 (13.3)	0	0

FDS, flexor digitorum superficialis; FDP, flexor digitorum profundus; FDM, flexor digiti minimi; FP, flexor pollicis; ED, extensor digitorum; EI, extensor indicis; EPB, extensor pollicis brevis; EPL, extensor pollicis longus.

**Table 2:** Results of Motor Examination of the Affected Arm

Reimplantation Patient	Trapezius	Deltoid	Pectoralis	Infraspinatus	Biceps	Triceps	Wrist Movements	Finger Movements	Global MRC Score (of 35)
1	3	2	1	0	4	3	0	0	10
2	5	1	1	1	0	1	0	0	4
3	5	0	0	0	0	0	0	0	0
4	4	0	0	0	0	0	0	0	0
5	5	4	3	3	1	3	0	0	14
6	4	4	3	1	1	1	0	0	10
7	4	3	2	1	2	1	0	0	9
8	4	2	2	1	1	0	0	2	8
9	4	0	0	1	1	1	0	0	3
10	5	1	1	3	0	0	1	3	9
11	5	3	3	3	2	3	0	0	13
12	4	1	0	0	0	1	0	0	2
13	5	1	0	0	0	0	0	0	1
14	5	2	3	4	2	0	0	0	11
15	5	1	3	3	1	2	0	0	10

Values reflect Medical Research Council (MRC) grades.

### Clinical Assessment of Sensory Recovery

#### Dermatomal Distribution

All patients had a completely insensate arm, including C5 to T1 dermatomes, preoperatively. After surgery, of the 15 patients, 14 (93%) reported light touch and 13 (87%) pinprick in the C5 distribution. Eight (53%) had light touch sensation and 7 (47%) pinprick in the C6 territory. In C7 distribution, only 2 (13%) reported light touch, and 1 (7%) reported pinprick. One patient (7%) reported sensation in the C8 territory in both pinprick and light touch, and 8 of 15 (53%) patients had sensation in the T1 distribution in light touch and 7 (47%) in pinprick. Horner syndrome was reported in 6 of 15 reimplantation patients (40%).

Reduced temperature sensation was perceived in the region of the shoulder in 13 reimplantation patients, at the elbow in 7 subjects, and in no patients at the wrist. Vibration sensation was perceived at the shoulder in 12 patients, at the elbow in 3 subjects, and at the wrist in 2 patients. Proprioception was accurate at the shoulder in 13 reimplantation patients, at the elbow in 7 patients, and no patients had accurate proprioception at the wrist.

#### Abnormal Sensations: Tinel Sign

None of the patients a positive Tinel sign at the shoulder preoperatively. After surgery, Tinel sign on percussion in the region of the nerve roots at the base of the neck was present in 4 patients with reimplantation. In all 4 patients, percussion over the region of the nerve roots provoked paresthesia (pins and needles) in the whole upper arm and forearm. In addition, paresthesia also was elicited in the dorsal central aspect of the hand in one patient and in another subject in the thumb.

#### Referred Sensations

**Figure 1:**
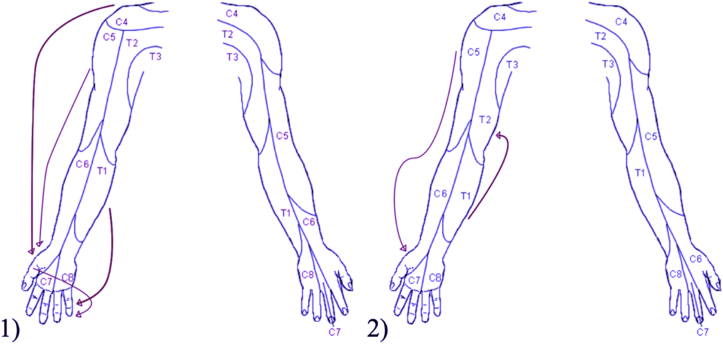
Schematic representation of referred sensation in 2 patients. 1) Sensation in the thumb and index finger on stimulating at the lateral aspect of the upper arm and sensation in T2 distribution on stimulating at the medial forearm. 2) Sensation in little finger when touching the thumb, sensation in little finger when inner aspect of forearm, sensation in the thumb on stimulating at C4 distribution, and sensation in the thumb on stimulating at C5 distribution.

Two patients described perception of sensations after reimplantation in remote locations not stimulated (Figure 1). Specifically, one patient perceived sensation in the little finger (C8 distribution) on testing sensation at the thumb (C6) and medial aspect of the forearm (T1 dermatome). In addition, the same patient reported sensation in the thumb on stimulating at the base of the neck (C4 dermatome) and at the lateral aspect of the upper arm (C5 dermatome). A different patient reported sensation in the medial aspect of axilla (T2) on stimulating the medial forearm (T1). Another patient reported pins and needles in the lateral aspect of the elbow on testing soft touch of C5 distribution with a cotton wool away from the region felt.

#### Pain and Allodynia

Of the 15, 5 patients reported pain in the hand in general, 1 in the palm, 2 in the dorsal aspect of the hand, 4 in the thumb or index finger (C6 dermatomal distribution), 1 in the fifth finger, 1 in the forearm, 2 in the whole arm in general, and 1 reported very mild pain or no pain. Interestingly, of the 4 who reported pain in the thumb or index finger, 3 had normal or reduced sensation in the C6 distribution.

The average VAS score reported 5.4 (SD, 2.03; range, 2–8). A Spearman correlation coefficient did not demonstrate a statistical significant correlation between VAS score and global MRC score (rho = 0.42; *P* = 0.11) or a correlation between VAS score and number of dorsal roots with sensory recovery (of 5; C5 through to T1) (z = −0.12; *P* = 0.67). Similarly, no significant relationship was detected between VAS score and time to the current assessment (rho = 0.23, *P* = 0.40).

One patient reported hyperesthesia (touch caused sensation of pain) in what was assumed to be the T1/T2 border on light touch examination, after reimplantation surgery. Two patients reported hyperesthesia in the C5 territory on soft touch and pinprick testing.

### Results of Patient-Reported Outcome Measures

The mean DASH score was 47.17 (SD, 22.98; range, 12–89). At the time of the interview, 9 of 15 patients (60%) were still working. Nine of the total 15 patients completed the DASH work questionnaire, mean score of 47.17 (SD, 22.98; range, 12.5–89.2). Regarding sporting activities, 8 (53%) of the 15 reimplantation patients completed the DASH sport questionnaire and 6 (40%) engaged in sporting activities. The group mean was 53.12 (SD, 38.23; range, 12.5–100). The mean Michigan Hand Outcomes Questionnaire total score was 25.676 (SD, 15.17; range, 1.43–54.29), and the mean score for the SF-36 physical health summary was 39.06 (SD, 9.69; range, 23–53) in the SF-36 physical healthy summary and 42.27 (SD, 14.82; range, 16–64) in the SF-36 mental health summary.

### Results of Nerve Conduction Studies

Of the 15 subjects, compound muscle action potentials (CMAPs) were recorded after reimplantation surgery in one patient on stimulation of both the median and ulnar. The median nerve demonstrated prolonged distal motor latency of 33.7 milliseconds, decreased CMAP amplitude of 3.5 mV and 3.1 mV when stimulated at the wrist and elbow respectively, and prolonged F-wave latency of 33.7 milliseconds. The ulnar nerve demonstrated normal distal motor latency of 2.7 milliseconds, decreased CMAP amplitude of 6.6 mV, 3.9 mV, and 3.7 mV when stimulated at the wrist, below the elbow, and above the elbow, respectively, and prolonged F-wave latency of 31 milliseconds.

Two patients showed evidence of sensory nerve action potentials (SNAPs) when stimulated at the index and middle finger and recorded at the wrist. Five patients showed SNAPs when stimulated at the palm and recorded at the wrist, including the 2 patients with responses from the index and middle finger. The same 2 patients also showed ulnar nerve conduction velocities when stimulated at the little finger and recorded at the wrist. All SNAPs recorded were of reduced amplitude and increased latency. None of the reimplantation patients showed evidence of response to radial nerve stimulation.

### Results of Needle EMG Studies

The results of the needle EMG studies are given in Table 3. Evidence of innervation of upper limb muscles was observed in total of 8 of the 12 patients examined (67%). MUAPs were detectable from the deltoid muscle in 6 patients, from the biceps in 7 subjects, from the triceps in 5, infraspinatous in 5, and first dorsal interosseous in 1. In addition, distant to the needle, MUAPs were observed in FDS in another patient. Furthermore, there was neurophysiological evidence of ongoing reinnervation with polyphasic nascent units in 3 patients in the deltoid muscle, 5 in the biceps, and 4 in the triceps and infraspinatous muscle.

**Table 3:** Raw Needle EMG Data of the Reimplantation Group

Muscle	Fibri/PSW	Duration	Amplitude	Polyphasicity	Recruitment	Interference
Deltoid						
Reimplant 1	–	–	–	–	–	–
Reimplant 2	3	↑	↑	Normal	Reduced, large units recruiting early	Severe decrease single units
Reimplant 3	3	–	–	–	No MUAPs	–
Reimplant 4	2	–	–	–	No MUAPs	–
Reimplant 5	–	–	–	–	–	–
Reimplant 6	2	↑↑	Normal	↑↑	Nascent units, reduced	Moderate decrease
Reimplant 7	2	↑↑	↑↑	↑↑	Reduced, large units recruiting early	Moderate decrease
Reimplant 8	2	↑↑	↓↓	↑↑↑	Nascent units, reduced	Severe decrease single units
Reimplant 9	2	–	–	–	No MUAPs	–
Reimplant 10	3	–	–	–	No MUAPs	–
Reimplant 11	3	↑	Normal	↑↑	Nascent units, reduced	Moderate decrease
Reimplant 12	3	–	–	–	No MUAPs	Moderate decrease
Reimplant 13	2	↑	↑	Normal	Reduced, large units recruiting early	Severe decrease single units
Reimplant 14	2	–	–	–	No MUAPs	
Reimplant 15	0	–	–	–	No MUAPs	
Biceps						
Reimplant 1	–	–	–	–	–	–
Reimplant 2	3	↑	↑↑	↑	Reduced, large units recruiting early	Severe decrease single units
Reimplant 3	3	–	–	–	No MUAPs	
Reimplant 4	–	–	–	–	–	–
Reimplant 5	–	–	–	–	–	–
Reimplant 6	2	↑	↑	↑	Reduced, large units recruiting early	Severe decrease single units
Reimplant 7	3	↑↑	Normal	↑↑	Nascent units, reduced, large units recruiting early	Severe decrease single units
Reimplant 8	2	↑↑	↓↓	↑↑↑	Nascent units, reduced	Severe decrease single units
Reimplant 9	2	–	–	–	Distant single units	–
Reimplant 10	3	↑↑↑	↓	↑↑↑	Nascent units, reduced	Severe decrease single units
Reimplant 11	2	–	–	–	No MUAPs	–
Reimplant 12	3	–	–	–	No MUAPs	–
Reimplant 13	3	–	–	–	No MUAPs	–
Reimplant 14	2	↑	↑	↑↑	Nascent units, reduced, large units recruiting early	Severe decrease single units
Reimplant 15	3	Normal	↓	↑↑	Nascent units, reduced	Severe decrease single units
Triceps						
Reimplant 1	–	–	–	–		
Reimplant 2	3	↑	↑↑	↑	Reduced, large units recruiting early	Severe decrease single units
Reimplant 3	3	–	–	–	No MUAPs	–
Reimplant 4	–	–	–	–	–	–
Reimplant 5	–	–	–	–	–	–
Reimplant 6	2	↑	↑	↑	Reduced, large units recruiting early	Severe decrease single units
Reimplant 7	3	↑	↓	↑↑	Nascent units, reduced, large units recruiting early	Severe decrease single units
Reimplant 8	2	–	–	–	No MUAPs	–
Reimplant 9	2	–	–	–	No MUAPs	–
Reimplant 10	3	↑↑↑	↓	↑↑↑	Nascent units, reduced	–
Reimplant 11	2	–	–	–	Nascent units, distant units	–
Reimplant 12	3	↑↑	↓	↑↑↑	Nascent units, reduced	Severe decrease single units
Reimplant 13	3	–	–	–	No MUAPs	–
Reimplant 14	2	–	–	–	No MUAPs	–
Reimplant 15	3	–	–	–	No MUAPs	–
Infraspinatus						
Reimplant 1	–	–	–	–	–	–
Reimplant 2	–	–	–	–	–	–
Reimplant 3	–	–	–	–	–	–
Reimplant 4	–	–	–	–	–	–
Reimplant 5	–	–	–	–	–	–
Reimplant 6	–	–	–	–	–	–
Reimplant 7	–	–	–	–	–	–
Reimplant 8	–	–	–	–	–	–
Reimplant 9	–	–	–	–	–	–
Reimplant 10	3	↑↑	↓	↑↑	Nascent units, reduced	Severe decrease single units
Reimplant 11	2	↑	↑↑	Normal	Nascent units, reduced, large units recruiting early	
Reimplant 12	–	–	–	–	–	–
Reimplant 13	–	–	–	–	–	–
Reimplant 14	2	Normal	Normal	↑↑	Nascent units, reduced	Severe decrease single units
Reimplant 15	3	Normal	Normal	↑↑	Nascent units, reduced	Severe decrease single units

EMG, electromyelography; PSW, positive sharp waves; MUAPs, motor unit action potentials; ↑, increased; ↓, decreased.

All patients demonstrated fibrillation potentials/PSWs. A Spearman correlation coefficient did not reveal a correlation between the extent of fibrillation potentials/PSWs and time since the reimplantation procedure in the deltoid, infraspinatous, biceps, or triceps (rho = −0.11, *P* = 0.72; rho = −0.89, *P* = 0.11; rho = −0.17, *P* = 0.59; rho = −0.17, *P* = 0.59, respectively). In addition, there was no significant correlation between time since the reimplantation and grade of reinnervation for deltoid, infraspinatous, biceps, or triceps (rho = 0.15, *P* = 0.63; rho = −0.08, *P* = 0.86; rho = 0.033, *P* = 0.92; rho = −0.011, *P* = 0.97, respectively).

Early recruitment of large units was noted in deltoid muscle in 3 patients, in the biceps in 4 patients, triceps in 3, and infraspinatous in 1.

## Discussion

In this study, we assessed 15 patients who had complete BP avulsion and reimplantation surgery. This is the first study to investigate the effects of reimplantation surgery in the chronic phase of recovery and demonstrate significant clinical and electrophysiological improvement. Patients who have had reimplantation surgery showed improved power in the deltoid, pectoralis, and infraspinatous muscles and global MRC score. Reinnervation by EMG criteria was found in infraspinatous, biceps, and triceps muscles. Distal muscle MUAPs were recorded from FDS in one patient and first dorsal interosseous in another. There was neurophysiological evidence of ongoing reinnervation with polyphasic, small-amplitude MUAPs (nascent units) in 3 patients in the deltoid muscle, 5 in the biceps, 4 in the triceps, and 4 in the infraspinatous muscle.

Greater motor recovery was shown in the proximal arm muscles, as expected since C5–C7 ventral roots were reimplanted. Regenerating axons reinnervate the most proximal muscle target that they encounter. The number of nerve root reimplantations is limited by the area of the spinal cord available for reimplantation, and the additional exposure required may increase technical complexity and therefore potential complication rates. Muscle recovery also is affected by the degree of muscle atrophy and fibrosis that occurs in chronically denervated muscles.^25^ All our patients received an intensive 2-week rehabilitation program in the immediate postoperative period with highly specialized rehabilitation services that aimed to increase and maintain range of movement, mobilize tight scar tissue, maintain good joint position and posture, adjust analgesics, and encourage return to activities in our unit. After this initial rehabilitation period, patients were referred to their local services. Consequently, rehabilitation programs varied significantly depending on local specialist availability, introducing variability in the data.

In addition, the time from injury to reimplantation is a further determining factor of the degree of motor recovery. It is believed that delays of more than 4 weeks significantly limit recovery, as avulsed roots and intertebral foramina become surrounded by scarred tissue. Because histologic examination to demonstrate regeneration of nerve fibers via the reimplanted roots was not possible, we aimed to minimize confounding of reinnervation via collateral sprouting from cranial or caudal nerve roots by only including patients with complete BP avulsion. Previous published cohorts include patients with varying numbers of root injuries. In our group of patients, any reinnervation would have to be supplied via one or more reimplanted roots.

Sensory testing revealed better recovery at C5 dermatomal distribution with less recovery of C6 and T1. The return of sensory function in avulsed dermatomes is difficult to explain, as only the ventral roots have been reconnected to the spinal cord. In addition, during the reimplantation procedure, the dorsal root ganglia often are excised. As in the ventral roots, sensory recovery could be attributed to collateral sprouting of fibers from an overlapping dermatome rather than repair through regeneration.^6^ This could explain recovery at the C5 and T1 dermatome from C4 and T2 collateral sprouting respectively. Alternatively, new processes could extend from dorsal horn neurons along the implanted ventral root, and regeneration of local interneurons also may play a role. The exact mechanism by which sensory recovery occurs is not yet understood.

Initial clinical observations have suggested that pain in the affected arm is related to the number of avulsed roots.^26^ It is, therefore, unsurprising that our patients in whom all 5 dorsal roots of the BP were avulsed experienced severe neuropathic pain in the whole arm. Clinical observations also suggested that successful surgical repair was associated with relief of avulsion pain and that improvement in pain severity was correlated with the return of muscle activity.^8^, ^12^, ^26^ In our study, a statistical significant correlation between pain and motor recovery or a temporal relationship from the time of surgery was not demonstrated.

There are some fundamental differences between our study and previous investigations of pain phenomena in BP injury. First, subjects studied in the previous reports included patients with any type of BP injury, including ruptures and avulsions of only 2 or more spinal roots involved which, consequently, resulted in a greater number of patients. To avoid such variability in our study, we examined a more homogeneous group of patients with complete BP injury alone. It is possible, therefore, that differences in the findings may due to either the exclusion of more modest injuries or failure to demonstrate a difference with our small sample size. However, it is not uncommon for patients to say that they have noticed improvement in pain after surgery but rather than a decrease in the absolute level of pain, they describe a subjective improvement in the distribution or quality of the pain.

Two reimplantation patients perceived referred sensations. Referred sensations have been described in a number of conditions, including amputation, somatosensory deafferentation, and in BP injury.^12^, ^26^ The pathophysiology of such sensations is also poorly understood; however, one proposed mechanism is through sprouting of primary afferent nerve fibers within the gray matter of the dorsal horn of the spinal cord. McMahon and Kett-White et al.^27^ have shown that primary afferent nerve fibers show greatly enhanced sprouting when 1) vacant synaptic sites are created by the degeneration of other primary afferent fibers and 2) the peripheral branches of the axons are actively regenerating. In our 2 patients with referred sensations, sensation was perceived in dermatomes immediately adjacent to the dermatome examined, suggesting that recovery occurs by collateral sprouting. The theory of collateral sprouting is supported in 2 patients who had a positive Tinel signs referred to the posterior aspect of the hand and thumb, and sensation also was present when the dermatomes of the hand and thumb were tested.

## Conclusions

We have demonstrated clinical improvements in outcome after BP reimplantation surgery as a treatment for complete BP avulsion. This is particularly true of neurologic recovery in the proximal arm muscles. Our results are the first to describe analytically the clinical recovery in patients with complete avulsion injury and reimplantation. Although a significant improvement in quality of life was not demonstrated, our study demonstrates a proof of principle that BP reimplantation surgery can result in reinnervation with neurologic recovery in this cohort of patients. Further improvement in functional recovery may be achieved by fine-tuning the surgical technique, performing surgery as early as possible, potentially by adding reparative cells, using neuroprotective and neuromodulating pharmacological agents such as minocycline and riluzole, and incorporating standardized rehabilitation protocols.^3^, ^28^ We are presently exploring the role for reparative cell therapies as an adjunct to BP reimplantation.^3^
